# 4471 Interactions of the Infant Nasopharyngeal Microbiota and Subjects’ Clinical Traits in Development of Viral Upper Respiratory Tract Infections and Acute Otitis Media

**DOI:** 10.1017/cts.2020.179

**Published:** 2020-07-29

**Authors:** Kamil Khanipov, George Golovko, Anna Nia, Lorraine Evangelista, Yuriy Fofanov

**Affiliations:** 1 University of Texas Medical Branch

## Abstract

OBJECTIVES/GOALS: Identify the interactions between nasopharyngeal bacterial pathogens, commensals, and patient clinical characteristics in relation to the development of viral upper respiratory tract infections (URI) and acute otitis media (AOM) in infants. METHODS/STUDY POPULATION: The subjects were part of a prospective, longitudinal study (2008–2014) of infants to evaluate the prevalence and risks for the development of URI and AOM. Healthy infants (n = 362) were enrolled from near birth and followed to the first episode of AOM up to 12 months of age. Nasopharyngeal specimens and clinical traits were collected at monthly intervals between 1-6 months, month 9, and during viral URI episodes. Subjects were closely followed for AOM development. 16S rRNA sequencing was performed on the nasopharyngeal swabs to identify their bacterial composition. Multidimensional (2, 3, and 4 dimensional) co-presence, co-exclusion, and one-way relation patterns were identified between the microbiome compositions, health status, and other collected clinical traits. RESULTS/ANTICIPATED RESULTS: We analyzed 971 specimens collected monthly and during URI and AOM episodes from 139 infants. Of the 139 enrolled subjects, 96% had 2 or more healthy samples, 77% contracted URI/AOM during the study period, and 60% had at least 1 healthy sample before URI/AOM onset. Otopathogens (Moraxella, Haemophilus, and Streptococcus), Staphylococcus, and Pseudomonas were the most common pathogenic genera. Corynebacterium, Dolosigranulum, and Acinetobacter were 3 most abundant commensal bacterial genera. Samples from infants with AOM in the first year had a significantly higher relative abundance of Haemophilus, Enterobacter, and Yersinia, and lower relative abundance of Corynebacterium, and Pseudomonas compared to samples from infants who did not develop AOM. DISCUSSION/SIGNIFICANCE OF IMPACT: Identification of complex multidimensional interaction patterns within microbial communities and environmental factors is vital to understanding disease onset risk and prevention. Prophylactic microbiome and environmental factor modulation between enterotypes could be used to reduce URI/AOM onset in infants.

